# Diagnosis and management of superficial arteriovenous malformations: French healthcare network’s recommendations

**DOI:** 10.1186/s13023-024-03413-5

**Published:** 2025-01-30

**Authors:** Olivia Boccara, Didier Salvan, Claude Laurian, Caroline Degrugillier-Chopinet, Nathalie Degardin, Jean-Guillaume Dillinger, Julie Malloizel-Delaunay, Stéphane Mouton, Stéphane Munck, Annabel Maruani, Annouk Bisdorff-Bresson

**Affiliations:** 1 French Coordinator Reference Center for Superficial Vascular Anomalies in Children and Adults of FAVA-Multi Network, Paris, France; 2 Department of Dermatology, University Hospital Necker-Enfants Malades, Paris, France; 3 FIMARAD Network, Paris, France; 4 Department of Otorhinolaryngology, Lariboisière Hospital, Assistance Publique-Hôpitaux de Paris (APHP), Paris, France; 5 Department of Vascular Surgery, Saint Joseph Hospital, Paris, France; 6 Department of Clinical Physiology and Echocardiography, Heart Valve Clinic, Lille University Hospital Center, CHU Lille, Lille, France; 7 Department of Pediatric Plastic Surgery, Centers of Competence for Facial Clefts and Deformities (MAFACE) & Pierre Robin Syndromes and Congenital Sucking-Swallowing Disorders (SPRATON), Timone-Enfant Hospital, Marseille, France; 8 Cardiology Department, Centre de Référence et d’Education aux Antithrombotiques d’Ile de France (C.R.E.A.T.I.F.), Lariboisière Hospital, APHP, Paris, France; 9 Department of Vascular Medicine, Rangueil Hospital, CHU de Toulouse, Toulouse, France; 10 Department of Teaching and Research in General Practice, University Côte-d’Azur, Nice, France; 11 INSERM 1246-SPHERE, Department of Dermatology, Center of Reference for Rare Vascular Diseases MAGEC-Tours, University of Tours, Tours, France; 12 Department of Neuroradiology, Lariboisière Hospital, APHP, Paris, France

**Keywords:** Superficial arteriovenous malformations, Differential diagnosis, Assessment of disease extent, Medical and therapeutic management, Psychosocial care, French Healthcare organization

## Abstract

Superficial arteriovenous malformations are rare fast-flow lesions. They consist of arteriovenous shunts, without cellular hyperplasia or proliferation, which develop in the surrounding tissues (cutaneous, subcutaneous, muscular, bone). Although benign, they are among the most severe of superficial malformations. Their evolution can be life-threatening in exceptional cases. With the aim of optimizing diagnosis and management worldwide, this  protocol offers a state of the art for the diagnosis and management of these diseases. To this end, the French healthcare network specialized in these diseases have drawn on literature data and experience. Developed from the French National Diagnosis and Care Protocol, it presents the patient journeys for initial and differential diagnoses, and personalized therapeutic strategies. This requires a multidisciplinary team, with specialized professionals in handling genetic, treatment and psychosocial issues.

## Summary of the PNDS intended for the general practitioner

### Definitions

Superficial (extra-cerebral) vascular malformations are classified according to vascular flow velocities as either fast or slow flow.

Superficial arteriovenous malformations (sAVMs) are fast-flow lesions. They consist of arteriovenous shunts, without cellular hyperplasia or proliferation, which develop in the surrounding tissues (cutaneous, subcutaneous, muscular, bone).

sAVMs are rare and benign, but among the superficial vascular malformations, they are the most severe, as their evolution can be life-threatening in exceptional cases.

These lesions are congenital, for the most part sporadic, occasionally familial, and located primarily in the cervicofacial region, followed by the limbs and trunk.

They may sometimes be associated with other tissue anomalies, resulting in syndromic forms.

Subcutaneous forms are rarely diagnosed in childhood, but a warm, pulsating “false” port wine stain should prompt referral to a specialist. These lesions exist since birth, but most often become symptomatic after a microtrauma (e.g. direct shock) or hormonal change (puberty, estrogen-progestogen oral contraception or pregnancy).

### Diagnostic management

**Fig. 1:**
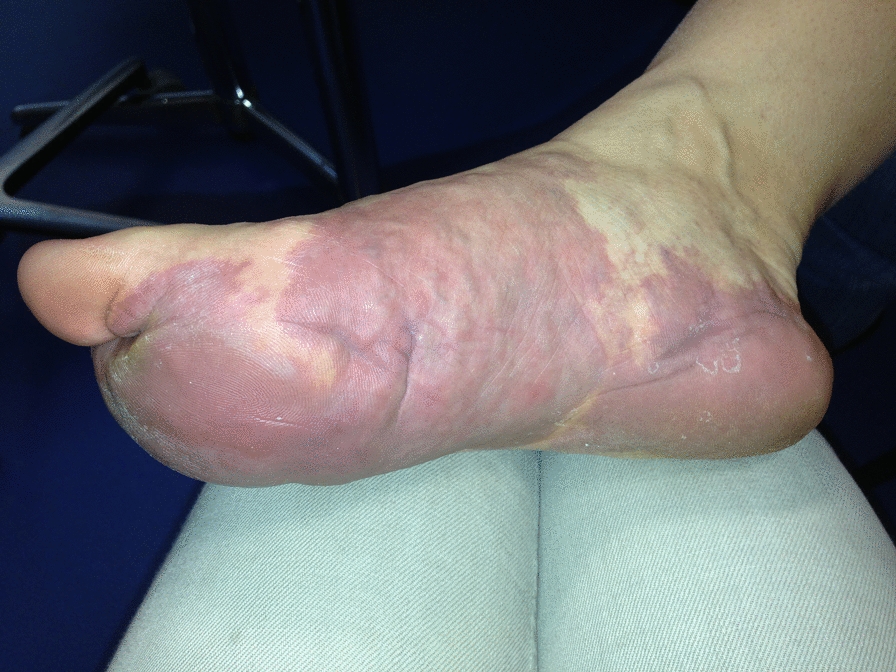
“False” port-wine stain: too warm, raised, atypical location, pulsating…

A sAVM should be suspected in the presence of a reddish swelling/spot with an increase in skin temperature during clinical palpation, and/or an atypical localization or clinical appearance, unlike a simple port-wine stain (Fig. 1).

The following can be found:

- A pulsatility, a « thrill» (vibration),
- Hemorrhage,
- Asymmetry or discrepancy in limb length and/or girth.

The first additional examination to be conducted is a Doppler ultrasound, performed by an experienced technician to confirm the diagnosis of a sAVM.

Diagnosis is essential, as any inappropriate treatment (pulsed dye laser treatment, partial surgery, proximal embolization, unsuitable oral contraception, etc.) can stimulate the sAVM and make it more clinically aggressive: enlargement, hemorrhage, pain, ulceration, necrosis, heart failure.

Any patient with a sAVM should be referred to a specialized multidisciplinary team, where second-line examinations (MRI, other explorations) will be requested, in order to assess the regional and general impact of the AVM and for pre-therapeutic purposes.

Given the bleeding risk and sometimes the AVM expansion after biopsy, the indication for this procedure is limited (suspicion of a malignant tumor). As somatic mutations have been detected in sAVMs, it has however become more frequent. As a result, the use of targeted treatment strategies is now more frequently considered.

### Differential diagnoses

The main differential diagnoses concern other vascular malformations, which are of slow flow (capillary, venous and lymphatic malformations), and hypervascular tumors (infantile hemangiomas, intramuscular capillary-type hemangiomas, malignant tumors such as certain soft-tissue sarcomas, PTEN hamartoma of soft tissue (PHOST), fibroadipose vascular anomaly (FAVA), etc.).

### Therapeutic management

sAVM management is always complex and requires multidisciplinary consultation. The natural history is characterized by either a gradual increase in size or intermittent exacerbations triggered by hormonal changes or trauma. Therapeutic strategies may include clinical follow-up, compression garments, embolization and/or excision surgery and drug therapies.

Only complete surgical excision should be considered curative, and it is only possible for localized sAVMs. Some patients present a significant risk of intraoperative bleeding which requires preoperative treatment planning.

#### Psychological management

Psychological support must be offered to sAVM patients and their caregivers.

#### Social aspects

Several forms of social assistance are available for adults and children suffering from a disabling sAVM. Patients/parents should contact their general practitioner, a specialist from the hospital's multidisciplinary team, or the hospital's social services department.

#### School and leisure activities

It is important for patients to maintain their physical or sporting activities, hobbies and professions, but these must be appropriate, i.e. to avoid direct trauma to the sAVM. Contact sports or sports that could lead to a direct blow to the sAVM are contraindicated.

### Follow-up of patients

In the majority of cases, patients are informed by their doctor that their sAVM is chronic and not completely curable. A professional multidisciplinary meeting states on a therapeutic strategy, which is then proposed to the patient.

Regular follow-up is proposed, at intervals ranging from once every three months to once every 2 years.

Additional tests (imaging, cardiac workup and others, depending on sAVM location) may be requested to detect any potential complications.

#### Contraception

Estrogen-based contraception is contraindicated, as it may induce a progressive exacerbation of AVMs. There are no particular restrictions on other types of contraception (progestative).

#### Pregnancy

Pregnancy is a period of hormonal change that can induce an exacerbation of the sAVM. However, in the majority of cases it is not contraindicated and a close clinical and Doppler ultrasound follow-up is mandatory.

#### Genetic counseling

Isolated AVMs are not inherited. Somatic mutations in genes involved in the RAS/MAPK signaling pathway have been recently identified.

There are, however, very specific syndromic and familial forms of autosomal dominant transmission: the "capillary malformation-arteriovenous malformation" syndromes. They are characterized by small capillary malformations, sometimes punctiform (papular) telangiectasias and a superficial or intracranial and intra medullary AVM in < 30% of cases. The genes involved are *RASA1* and *EPHB4*.

### Contacts and general resources

- Reference Centers* and Competence Centers of the FAVA multi rare disease network

* A Reference Center brings together a highly specialized hospital team with proven expertise in a rare disease—or group of rare diseases—and which develops activities in the fields of care, teaching/training and research.

Consult the link for a list of the network’s centers/sites: https://favamulti.fr/filiere-fava-multi/organisation-de-la-filiere/

It includes the Coordinator Reference Center for Superficial Vascular Anomalies in Children and Adults of FAVA-Multi Network, Paris, France: https://www.malformations-vasculaires.fr/

- The Psychological Platform—Psy FAVA-Multi:

Psychological help adapted to rare vascular disease problematics is provided by FAVA-Multi. With a dedicated email address: psy.favamulti.bch@aphp.fr

For more information, consult the link for: https://favamulti.fr/parcours-patient/aide-psychologique/

- Orphanet and European Joint Program Rare Diseases VP-Portal

Consult the links for

- Orphanet, the French portal for rare diseases and orphan drugs:http://www.orpha.net
- European Joint Program Rare Diseases VP-Portal: https://vp.ejprarediseases.org/discovery

Information and resources on superficial arteriovenous malformations can be found under the following ORPHA codes:

- ORPHA: 211266 Rare arteriovenous malformation
- →ORPHA: 156230 Facial arteriovenous malformation
- →→ORPHA: 141174 Mandibular arteriovenous malformation
- →→ORPHA: 141168 Frontonasal arteriovenous malformation
- →→ORPHA: 141171 Maxillary arteriovenous malformation  
- ORPHA: 98731 Congenital arteriovenous fistula 

## Background and objectives

Superficial (extra-cerebral) vascular malformations are classified according to vascular flow velocities as either fast or slow flow.

**Fig. 2:**
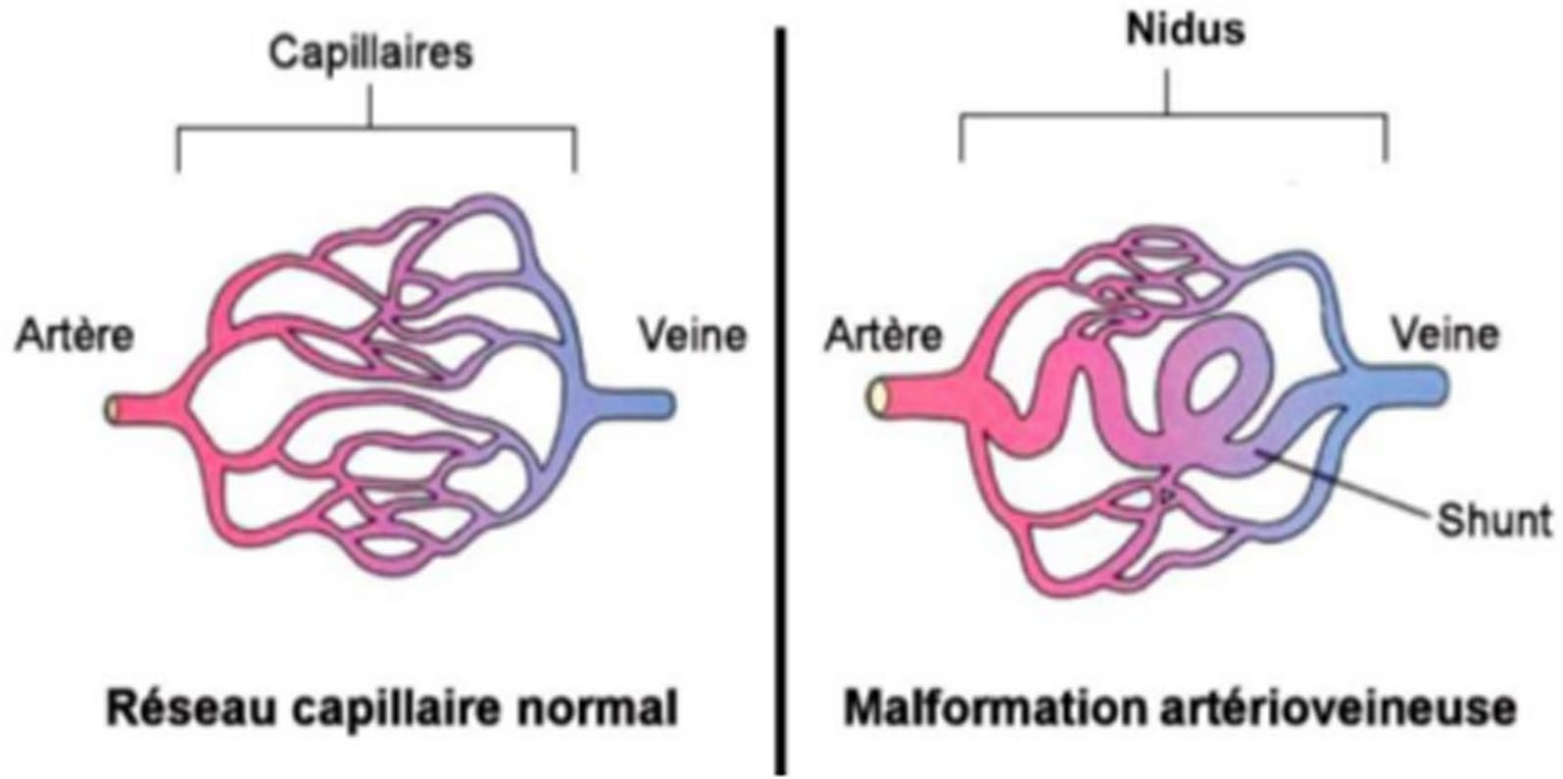
Arteriovenous shunt in sAVM, from PNDS “Les malformations artério-veineuses superficielles” 2021 [1]

Superficial arteriovenous malformations (sAVMs) are fast-flow lesions. They consist of arteriovenous shunts (Fig. 2), without cellular hyperplasia or proliferation, which develop in the surrounding tissues (cutaneous, subcutaneous, muscular, bone).

sAVMs are rare and benign, but among the superficial vascular malformations, they are the most severe, as their evolution can be life-threatening in exceptional cases.

These lesions are congenital, for the most part sporadic, occasionally familial, and located primarily in the cervicofacial region, followed by the limbs and trunk.

They may sometimes be associated with other tissue anomalies, resulting in syndromic forms.

Subcutaneous forms are rarely diagnosed in childhood, but a warm, pulsating “false” port wine stain should prompt referral to a specialist. These lesions exist since birth, but most often become symptomatic after a microtrauma (e.g. direct shock) or hormonal change (puberty, estrogen-progestogen oral contraception or pregnancy).

The purpose of this manuscript is to present worldwide the state of the art for the diagnosis and therapeutic management of these diseases. Intended for concerned medical and paramedical professionals, it explains the optimal care pathway of a patient with a given superficial AVM. Developed from the French National Diagnosis and Care Protocol (PNDS), its aim is to optimize and harmonize the management and monitoring of this rare disease in France. It also point out medicinal products used for indications not covered by their Marketing Authorization (MA), as well as medications, products or services required for patient care but not usually reimbursed by health insurance.

The review can be notably used as a reference document by the treating physician in relation with the specialized physician, particularly when establishing the patient’s care protocol and application for exemption from medical expenses.

It cannot, however, consider all specific cases, all comorbidities or complications, all therapeutic particularities, all hospital care protocols, etc. It does not claim to provide an exhaustive list of possible management strategies, nor does it replace the physician’s individual responsibility to their patient.

The protocol does however describe the gold standard of management for a patient with a superficial AVM in 2023.

## Introduction

Superficial arteriovenous malformations (sAVMs) belong to the spectrum of superficial vascular anomalies, a heterogeneous group of superficial vascular tumors and malformations. In the classification proposed in 1982 by Glowacki and Mulliken [2] and accepted by the international group of experts from the *International Society for the Study of Vascular Anomalies* (ISSVA) in 1996 (then updated in 2018) [3].

AVMs are considered to be vascular malformations, not tumors. Hemodynamically, they are classified as fast-flow malformations, as opposed to the slow-flow malformations (capillary, lymphatic or venous malformations) (www.issva.org).

sAVMs should be distinguished from cerebral AVMs as they are extracranial, showing clear differences in terms of characteristics, clinical course and implications.

sAVM are rare malformations and their exact incidence is unknown.

They are composed of direct arteriovenous communications, without intermediate capillaries, known as 'shunts,' which form a nidus (Fig. 2). They differ from arteriovenous fistulas, characterized by a single communication between an artery and a vein, without a nidus. In AVMs communications are multiple.

sAVMs develop during the embryonic period as a result of abnormal vasculogenesis. In the vast majority of cases, they occur sporadically. Postzygotic somatic gene mutations have been identified in some cases, affecting the Ras/MAPK (*mitogen activated protein kinase*) signaling pathway. There are also specific syndromic and familial forms, where other germline mutations in the Ras pathway have been identified [4, 5].

sAVMs develop in a localized tissue structure (cutaneous or subcutaneous, sometimes muscular or osseous), and are only rarely diffuse. They most often occur in the cervico-facial area, but they can also affect the limbs (particularly the extremities), the trunk and, more rarely, intra-abdominal organs.

They are present at birth, but for the most part become symptomatic during childhood, particularly during puberty. They may, however, appear in adulthood.

They can progress from a stage of "quiescence" to a more aggressive stage with local or general complications. This can occur following a trauma or an hormonal stimulation (puberty, contraception or pregnancy).

## Clinical diagnosis and differential diagnoses

### Objectives

The diagnosis of sAVMs is suggested upon clinical examination and confirmed through imaging studies. The aim of the specialized consultation is to assess the diagnosis of sAVMs, to evaluate the local, regional, general and psycho-social consequences, and to propose appropriate management and treatment.

### Clinical presentations

#### Sporadic AVM

A sporadic sAVM is a single focal lesion, which may be segmental and affect any part of the integument. It is most likely to appear after a trauma, or during puberty, pregnancy or upon the initiation of combined estrogen-progestogen oral contraceptives. It may present as a warm, and/or pulsating mass. It may be covered by a lesion resembling to a capillary malformation ("false” port wine stain) consisting of dilated capillary vessels, whose contours are often not clearly defined. This “false” port wine stain is sometimes the only diagnostic element, particularly in small children, that preceeds the appearance of the underlying pulsating mass. Other presentations are possible on an extremity (ears, lips, fingers, toes, etc.): keratotic appearance, red or purplish, warm.

Clinical examination is used to assess expansion and locoregional complications: condition of the skin (dilated drainage veins, risk of cutaneous or mucosal ulceration), pain, functional impairment, risk of hemorrhage. It also evaluates general consequences, particularly cardiac. A cardiologic work-up is necessary in large sAVMs to detect heart output failure due to increased flow and arteriovenous (AV) shunting. It is also necessary to assess the socio-professional and psychological impact that the superficial AVM can have on the patient.

Finally, it is necessary to evaluate the degree of clinical treatment urgency to define the management and treatment strategy on a case to case basis.

#### Familial AVMs: CM-AVM

Certain forms are syndromic. One example is CM-AVM (capillary malformation-arteriovenous malformation) (type 1 and 2), which is inherited in an autosomal dominant manner. Diagnosis of this syndrome is based on the presence of small, well delineated capillary malformations (1 to > 10) that range from 0.5 to several centimeters in diameter, which are randomly distributed and surrounded by a peripheral halo. They may be congenital or present at a later stage in life. Punctiform telangiectasias surrounded by a peripheral halo may be present on the hands (Bier's spots) or in the perioral region (as seen in hereditary hemorrhagic telangiectasia). This presentation should prompt to a clinical argument for the presence of an AVM or arteriovenous fistula of a limb (limb enlarged/elongated and warm), in this case constituting Parkes-Weber syndrome, or an AVM of the central nervous system. The overall risk of an associated AVM (cerebral and /or spinal) is estimated at between 5 and 30%, for all AVMs combined. The search for the causal gene mutation can be carried out (mutations of the *RASA1* and *EPHB4* genes, identified respectively in CM-AVM type 1, which includes Parkes-Weber syndrome, and CM-AVM type 2) [4, 5].

### Stages of severity and evolution

The natural history of sAVMs is marked by an aggravation that may be very gradual, or may be sudden, triggered by an event such as puberty, pregnancy or physical trauma.

There are 4 stages of sAVM severity, with stages III and IV being potentially life-threatening (Schobinger classification, see Table 1). Stage I corresponds to a quiescent lesion. In Stage II the AVM expands and becomes a pulsating mass. At an advanced stage, trophic disorders such as ulceration may be observed in connection with venous overload (stage III), resulting in bleeding. When the flow is significant, cardiac complications may occur (stage IV), which can be life-threatening.

**Table 1:** Schobinger Classification (of AVM severity). The classification was introduced at the 1990 Congress of ISSVA

Stages of severity	Description
I	Dormant stage, quiescent lesion ± beating
II	Expansion stage, pulsatile lesion
III	Stage II + bleeding, necrosis, pain or superinfection
IV	Stage III + cardiac failure

### Professionals involved

The diagnosis is usually suggested by the general practitioner, pediatrician or dermatologist. A Doppler ultrasound is then performed by a vascular physician or radiologist, who will be able to confirm the diagnosis with greater certainty. The patient must then be referred to a specific multidisciplinary expert center, comprising a doctor (dermatologist/vascular doctor), a radiologist and a surgeon, who will decide together on the necessary investigations, the possible treatments and the monitoring procedures. The initial management can also be carried out by emergency physicians in the event of bleeding that marks the onset of the diagnosis.

Depending on the location and complications of the sAVM, other specialists may be called upon (orthopedic surgeon, pain specialist, cardiologist, geneticist, physiotherapist, psychologist, nurses, etc.).

### Differential diagnoses

The main differential diagnoses are superficial vascular tumors.

#### In infants

- Infantile hemangioma (benign vascular tumor) is the main differential diagnosis. The initial clinical appearance of a warm mass (particularly for subcutaneous infantile hemangiomas) can be misleading, but hemangiomas are much more frequent, present in children but not adults, and have different kinetics. The Doppler ultrasound shows a tissular component as well as arterial and venous flows, misleadingly suggesting the diagnosis of an AVM. However, its localized, tissue-specific, well-defined subcutaneous nature, combined with a characteristic clinical course, facilitates its diagnosis.
- Soft-tissue sarcoma (rabdomyosarcoma, angiosarcoma…), a malignant vascularized tumor, is a rare differential diagnosis that must be considered when faced with a hard, rapidly growing, vascular tumor.

#### In children and adults

- Pyogenic granuloma: small inflammatory lesion of rapid-onset, often appearing on skin after a trauma. They may be pedunculated, superficial with a surface described as “raspberry-like” in appearance, and bleed on contact.
- Spider angioma: cutaneous vascular anomalies, which are star-shaped and centered by a dot, with smaller ones often seen in children; they can be observed in adults with hepatocellular insufficiency
- Hereditary hemorrhagic telangiectasia: to be considered in the presence of multiple telangiectasias with arteriovenous fistulas in the ENT region, epistaxis and pulmonary, hepatic or cerebral AV fistulas/malformations (with a family history).
- Hypervascularized soft tissue malignancies: to be considered in the presence of any rapidly growing tumor with a vascular appearance.

Any vascularized tumor lesion may be mistaken for an AVM; however, the presence of a tissular lesion should raise the suspicion of a tumor, justifying referral to a specialized center.

### Diagnostic confirmation and assessment of disease extent: additional examinations

#### Confirmation of the diagnosis

Diagnosis is based on clinical and ultrasound findings. Other additional examinations (cross-sectional imaging: MRI and/or CT scan) are necessary to support the diagnosis and determine the locoregional extension of the sAVM.

#### Doppler ultrasonography

Doppler ultrasonography is a first-line imaging technique used in sAVM patients. It confirms a poorly defined lesion with fast-flow and arteriovenous shunting, resulting in very high flow velocities, elevated diastolic flow and low resistance index (< 0.5) which is useful in distinguishing other vascular malformations (low flow: lymphatic or venous) from a quiescent sAVM. Arterial flow dynamics measurement in the main proximal artery compared to the contralateral side can be helpful to evaluate the evolution of the sAVM. Although these measurements can be used to monitor sAVM progression, they are highly operator-dependent.

#### MRI and CT

MRI can be used to assess deep tissue involvement, and CT angiography in the arterial phase provides a good analysis of AVM angioarchitecture as well as any soft tissue and/or bone involvement.

Follow-up will be performed by a clinical exam and non invasive imaging modalities such as Doppler ultrasound and/or cross-sectional imaging.

MRI is preferred in children, as it is a non-irradiating imagining technique. Sedation is generally required in children under 6 years of age when performing an MRI.

#### Plain X-rays

Standard plain radiography of the affected limb is useful for searching for intraosseous localizations, when clinical suspicion exists.

Radiographic measurement of the limb should be requested to evaluate any lower limb length discreapancy.

#### Angiogram

Angiogram is not a first-line procedure. Its use should be discussed in a specialized multidisciplinary team meeting, as it may, in certain cases, be useful in deciding on sAVM treatment stategies. Its aim is to determine AVM angioarchitecture and the treatment approach.

#### Biopsy: histopathology and molecular analysis

Tissular biopsy is usually contraindicated in sAVMs, as it can cause periprocedural bleeding and trigger disease progression. It may be discussed and carried out by a specialized multidisciplinary team for the following purposes:

1. histopathological analysis—it will reveal arteriovenous shunts and the absence of tumor proliferation in cases of diagnostic doubt (particularly with a malignant tumor);
2. Molecular analysis—searching for somatic mutations in the genes *KRAS, NRAS, BRAF,* and *MAP2K1* (Table 2). Currently, this analysis is at the intersection of research and care, as targeted therapies are under investigation.
3. Analysis on surgical samples following excision surgery

**Table 2:** Studies on the identification of somatic mutations in sporadic AVMs

References	Number of subjects tested	Number of subjects with a somatic mutation	Genes
Lekwuttikarn et al. [6]	1	1	*MAP2K1*
Al-Olabi et al. [7, 8]	25	9	*KRAS, NRAS, BRAF, MAP2K1*
Lapinski et al. [9]	4	4	*RASA1*
Wooderchak-Donahue et al. [10]	69	60	*RASA1*
Couto et al. [11]	10	7	*MAP2K1*
Chakraborty et al. [12]	41	20	*MAP2K1, BRAF*
Amyere et al. [5]	54	47	*EPHB4*
Revencu et al. [4]	68	58	*RASA1*

Pilot studies are being performed to explore the feasibility of liquid biopsies in sAVMs.

#### Cardiology consultation

A cardiac workup is performed if there are symptoms (asthenia, dyspnea), clinical signs of heart failure or if a murmur is detected on auscultation. A cardiac work-up should also be requested if the sAVM is large or proximal. The aim is to identify high-cardiac outflow heart failure, characterized by elevated cardiac output (> 8 L/min at rest) with normal or supranormal ventricular function, associated with reduced peripheral arterial resistance. Indeed, the existence of a peripheral left to right shunt linked to the AVM leads to an increase in preload and a rise in cardiac output. Over time, to adapt to this additional workload, the heart hypertrophies and then dilates. Cardiac examinations must therefore be repeated over time in the case of large sAVMs. The frequency is adapted on a case-by-case basis by the specialized expert team.

The cardiac work-up usually includes an electrocardiogram and a transthoracic echocardiogram.

#### Other consultations

Depending on the location of the sAVM, further investigations may be required (consultations with specialists in ENT, ophthalmology, orthopedics, etc.).

#### Genetic counseling

As previously mentioned, around a half of sporadic AVMs have now been shown to result from a somatic, post-zygotic genetic mutation affecting the RAS-MAPK signaling pathway. As this genetic mutation is not germline, it is not inherited, so there is no risk of recurrence in the family. The identification of a somatic genetic mutation may help to determine the best course of medical treatment using specific inhibitors of the signaling pathway involved, which are currently being evaluated.

In CM-AVM, germline mutations *(RASA1* or *EPHB4*) can be identified. The mode of inheritance is autosomal dominant, meaning that the risk of passing this mutation on to offspring is 50%. However, the occurrence of an AVM (superficial and/or cerebral) is not systematic when a patient is a carrier of a causal mutation: the risk is less than 10% in the case of an *EPHB4* mutation, and around 20% for a *RASA1* mutation.

In the case of a couple in which one member is a carrier of the mutation, particular care is required during pregnancy. In the event of transmission to the fetus, rare cases of arteriovenous fistulas, especially in the brain, can lead to complications in utero and during the neonatal period.

#### Announcing the diagnosis and informing the patient

The diagnostic announcement should be made in a quiet environment, preferably in the presence of a third party or both parents in the case of a child.

Time should be taken to:

- Explain the diagnosis and pathophysiology of the sAVM.
- Inform about the risk of bleeding, and the need for local protection. Situations with a high risk of traumatic injury should be avoided (professions and sports activities for children: favor low-impact sports). Each case must be discussed according to the location of the sAVM. Advice is personalized.
- Inform patients of the need for multidisciplinary follow-up and regular monitoring by a specialized referral physician. Therapeutic strategies will then be considered.
- Provide guidance for genetic consulting and testing.
- Assess the psychological impact of the malformation on the patient and their family/caregivers (parents, spouse) and suggest a consultation with a specialist (psychologist or psychiatrist), depending on the state of the patient or their caregivers and their needs.
- Discuss social assistance: when the situation is complex, it is necessary to set up social support measures to make daily life easier for the patient and their caregivers. For school, a personal learning plan, which is a written document that specifies the adaptations needed for the child’s or adolescent’s life in the community, is often necessary.
- Inform patients and their caregivers about the existence of patient organizations.

## Therapeutic management

### Objectives

The management of sAVMs is multidisciplinary and personalized. It must be discussed on a case-by-case basis, according to the patient's clinical and imaging findings and his or her needs. Follow-up is essential, as needs may change over time. The key points are the following:

- Provide simple clinical and ultrasound follow-up in asymptomatic patients, or if treatment of the sAVM as a whole is considered difficult or incomplete.
- Avoid aggravating a pre-existing sAVM by informing the patient of treatments or situations at risk.
- Consider a "radical" treatment, the only curative treatment, when feasible.
- Complications such as esthetic concerns, pain, trophic disorders, functional impairment, cardiac involvement and a general deterioration in overall health are the main reasons for treatment. In the event of hemorrhagic complications, emergency hemostasis may be required.
- Assess the need for psychosocial care.

### Professionals involved

Multidisciplinary management may require the involvement of the following specialists, depending on the location of the superficial AVM:

- Dermatologist
- Interventional radiologist
- Vascular physician
- Surgeons: vascular, ENT and maxillofacial, orthopedic, plastic, ophthalmic, pediatriac
- Cardiologist
- Pediatrician
- Anatomopathologist
- Genetician
- Pain specialist
- Attending general practitioner
- Psychologist
- Social worker
- Nurse
- Physiotherapist
- Others…

### Medical management and treatment

#### Compression

Local compression is an integral part of the medical treatment of sAVMs. It should be introduced from an early age when the diagnosis has been made and when the location permits.

Compression garments must be custom-made, especially for children, and must be adapted to the patient's morphotype so as not to be harmful (e.g. “tourniquet effect”). Compression garments come in a variety of forms: ankle braces, knee braces, socks, stockings, tights, sleeves, and also in materials of different thicknesses (material used for compression on severe burn patients, etc.).

In children, compression garments should be replaced as they grow in order to avoid constriction.

Local compression not only protects fragile skin or dilated vessels from trauma, but also compresses dilated subcutaneous vascular networks (reducing the flow of AVMs and potentially slowing their development). It is in no way curative. The minimum compression class selected should be type 2 (ideally greater than 15–20 mmHg), using material similar to that used on severe burn patients. Flat-knitted fabric may be useful for the extremities.

The link to a helpful leaflet for prescribing compression can be found here: *Fiches de compression de la SFMV* (find them here: https://www.portailvasculaire.fr).

#### Pharmacological management

The pathophysiological explanation for sAVMs is an anomaly in vasculogenesis, linked to somatic mutations that modify angioblasts differentiation and proliferation.

Several drugs have been tested, always on a small numbers of patients, with encouraging results in some cases. To date, however, no validated drug treatment has clearly demonstrated a major and repeated benefit on sAVMs. No controlled therapeutic trials have been published in the literature. The treatments described below are based on the empirical experience of specialized expert teams. Some molecules are used because of their known anti-angiogenic properties, while others are used to treat the symptoms of the sAVM (pain, bleeding, hyperflow or heart failure).

#### *Beta*-blockers

There are very few articles in the literature on the use of beta-blockers in sAVMs: the use of cardioselective or non-selective beta-blockers is still debated [13] (Table 3).

**Table 3:** Study on beta-blockers in the treatment of AVMs

Reference	Type of study	Number of subjects	Age	Molecule, dosage and duration	Efficacy	Tolerance
Lu et al. [13]	Prospective / cohort study	1	19	Oral propranolol treatment for 5 months	Good	Good

Beta-blockers reduce symptoms and improve comfort, probably due to their hemodynamic properties. By reducing sAVM flow and arterial pressure, they attenuate pulsatility, hemorrhagic phenomena and venous overload, and may help limit the unfavorable evolution of sAVMs. Their efficacy also lies in their capacity for distal arteriolar vasoconstriction and reduced arterial tension, enabling better endothelial compliance.

The use of a beta-blocker very moderately decreases the blood flow through the AVM, but its benefit lies in reducing the venous overload downstream of the shunt.

Furthermore, when there is cardiac involvement due to the sAVM, the treatment is the same as that used for high-output heart failure. A cardiological consultation is necessary before initiating this treatment.

#### Thalidomide

The anti-angiogenic properties of thalidomide have been known for some 25 years. It acts on the VEGF pathway, but also on the TNF (tumor necrosis factor), FGF (fibroblast growth factor) and PDGF (platelet-derived growth factor) pathways [14–19]. It is used in hematology and in the treatment of hereditary hemorrhagic telangiectasia. Stabilization or even clinical improvements have been reported in soft tissue AVMs. Side effects include peripheral neuropathy, depression, somnolence and thromboembolic events. It is prescribed orally at a long-term dose of 50–100 mg/day (in adults). A recent prospective experimental observational study performed on 18 patients with a severely stage III sAVM refractory to conventional therapies showed thalidomide efficiency in all patients regarding chronic pain, bleeding and ulceration [14] (Table 4). Grade 3 complications were dose-dependent and included asthenia and erythroderma.

**Table 4:** Table of studies on thalidomide in the treatment of AVMs

References	Type of study	Number of subjects	Age	Dosage and duration	Efficacy	Tolerance
Ge et al. [19]	Prospective / Cohort study	28	40–85	100 mg thalidomide/dayDuring 4 months	71.4%	Good
Boon et al. [14]	Prospective/Cohort study	18	19–70	In 5 patients: initial dose of thalidomide of 50 mg/day, which was increased to 100 mg/day or 200 mg/day within 2weeksIn 13 patients: 50 mg/day of thalidomide alone or in combination withembolization	Good	Good (in low-dose regimen)

Thalidomide is highly teratogenic. It is therefore essential to combine this medication with a reliable form of contraception.

#### Sirolimus

Sirolimus (rapamycin) is an oral mTOR (mammalian target of rapamycin) inhibitor. It was developed in the 90 s for its immunosuppressive properties (for solid organ transplants); mTOR is a serine/threonine protein kinase regulated by phosphoinositide-3-kinase (PI3K) and AKT. When the PI3K/AKT/mTOR signaling pathway is activated, it stimulates protein synthesis, cell proliferation and angiogenesis/lymphangiogenesis. Genetic variations in certain genes involved in this pathway (*PTEN, PIK3CA, TIE2 and AKT*) lead to its permanent activation, resulting clinically in the development of mainly slow-flow venous or lymphatic malformations. In these indications, sirolimus improves painful inflammatory symptoms, sometimes with a reduction in the volume of the malformation.

Its use has been proposed in the management of sAVMs, with very mixed and only temporary results. Furthermore, there are very few publications on this subject in the literature (Table 5). In our experience, some lesions show partial and temporary improvement but then relapse [20].

**Table 5:** Studies on sirolimus in the treatment in vascular malformations

References	Type of study	Number of subjects	Age	Dosage and duration	Efficacy	Tolerance
Hammill et al. [21]	Prospective /cohort study	6	7 months- 14 years	10–15 ng/ml sirolimus for 3 years	Good	Good
Adams et al. [22]	Prospective/cohort study	61	0–31 years	10–15 ng/ml de sirolimus pendant 12 × 28 jours	Good	Good
Triana et al. [23]	Prospective/ cohort study	41	0.16-47Years	0.8 mg/m^2^/12 heures	Good	Good
Gabeff et al. [20]	Retrospective/Cohort study	10	2–12.5	Starting dose of sirolimus ranged from 0.6 to 3.5 mg/m2	5 partial response and 5 no response	All had side effects

The dose most often used in the pediatric population is 0.1 mg/kg/day in one or two doses, and between 1 and 3 mg/day in adults, to achieve a residual serum level of between 4 and 12 ng/ml [22, 23]. Side effects include mouth ulcers, digestive disorders, asthenia, headaches and, biologically, thrombocytopenia, lymphopenia, hypercholesterolemia and hypertriglyceridemia.

Drug interactions are numerous, and some can increase the serum levels of sirolimus, such as certain antibiotics or cytochrome P450 inhibitors (grapefruit juice).

#### Bevacizumab

Bevacizumab is a VEGF-inhibiting monoclonal antibody used in oncology [24] and administered parenterally. It has demonstrated efficacy for the treatment of hereditary hemorrhagic telangiectasia involving bleeding complications. It can also add in controlling large hepatic AVMs [25]. The signaling pathways involved in AVMs may lead to an increase in VEGF, but it is not necessarily a key effector. The VEGF pathway is probably not a major target for treatment. It is a rarely used treatment with limited efficacy in sporadic AVMs [16, 26].

#### Matrix metalloproteinase (MMP) inhibitors

MMPs stimulate the degradation of the vascular basement membrane, allowing the migration of endothelial cells to form new vessels and provide vessel wall stability. They have been described as abnormal in AVMs [27].

Their value in the treatment of AVMs has not been validated.

- Marimastat: described in a single case, as an adjuvant therapy to embolization. The dose was 30 mg/day over the long term, with an increase to 120 mg/day for over 12 years, and it was well-tolerated. This medication is used in oncology for lung and digestive cancers [28].
- Doxycycline: A series has been conducted in cerebral AVMs, but no studies have been conducted involving extracerebral locations [13, 29].

#### RAS pathway inhibitors

**Fig. 3:**
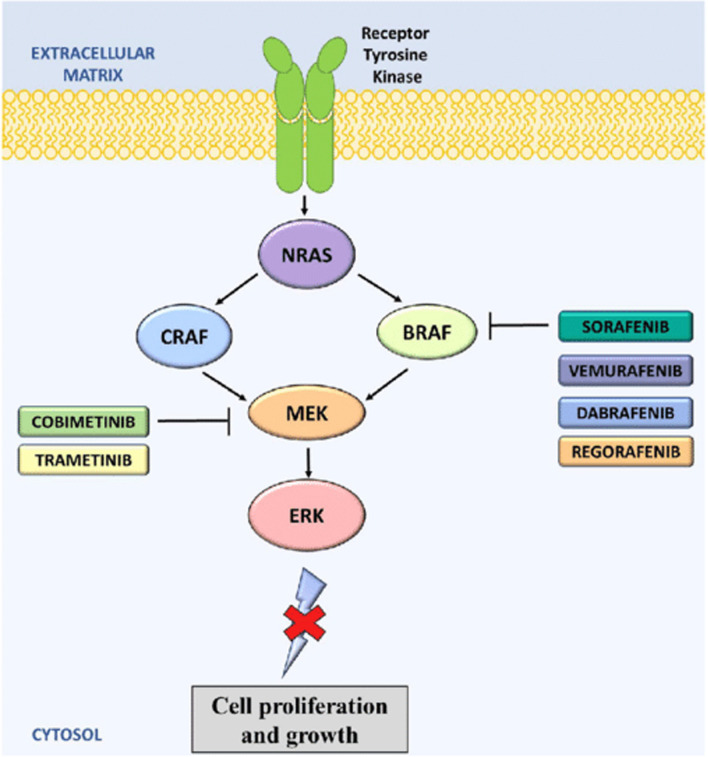
Diagram of the RAS/MAPK signalling pathway, extracted from article by Seebacher et al. 2019 (Fig. 5) [31]. RAS activates both the CRAF and the BRAF pathways. Shown BRAF and MEK inhibitors are FDA approved for the treatment of cancer

Given the identification of causal genetic mutations in RAS pathway genes, the use of specific inhibitors of this pathway, initially developed to treat cancers, is an option currently being explored (Fig. 3, Table 6). An international team has shown that trametinib, a MEK inhibitor, can reduce the size of an AVM in vivo in zebrafish embryos carrying a somatic RIT1 indel variants newly identified, and in one patient [30]. Therapeutic trials in humans may be further considered.

**Table 6:** Studies on anti-angiogenics in the treatment of sAVMs

References	Type of study	Number of subjects	Age	Molecule, dosage, duration	Efficacy	Tolerance
Lekwuttikarn et al. [6]	Prospective/Case report	1	11	Trametinib;0.5 mg once daily, then increased to 0.5 mg twice daily after one month	Good	Good
Edwards et al. [32]	Prospective/Case report	1	14 ?	Trametinib initiated at 50% of the goal dose for 2 weeks, then increased to 0.025 mg/kg daily	Good	Good
Nicholson et al. 2022 [33]	Prospective/Case report	1	16	Trametinib initiated at 2 mg daily for the first thirteen 28-day treatment cycles, then subsequently reduced	Good	Mitigated by dose reduction

### Surgical management

The treatment strategies and the techniques used differ according to sAVM location, its extent and its severity. The major difficulty lies in identifying the accurate moment to intervene, in relation to the stage of evolution of the AVM (Schobinger classification stages I to IV).

Surgical treatment must be discussed in a multidisciplinary consultation. The main guiding principles are:

(A) *Cases where the sAVM is not the therapeutic target* (large, extensive sAVMs, or location with limited surgical options, corresponding to the majority of patients with this malformation).

The targets are:

1. Orthopedic complications: limb length discrepancy with indications for epiphysiodesis for functional improvement;
2. Prevention of trophic complications or limit trophic disturbance: treatment with compression therapy and beta-blockers can prevent hemorrhagic complications and often leads to a prolonged status quo.
3. Monitoring of arterial complications upstream from the arteriovenous shunts (aneurysms due to increased flow velocities that require ultrasound monitoring and surgical correction in certain cases).

(B) *The target may be the nidus of the sAVM*

Surgery is recommended if the clinical presentation justifies it, and depending on whether the tissue involvement of the nidus is clearly identified as subcutaneous, intramuscular, or intraosseous.

If there isn't a precise identification of the tissues involved allowing for well-planned surgery (but still with risk), the surgery will have a high recurrence risk regardless of the AVM location.

#### Indications for surgery

When feasible, complete surgical resection of the sAVM (with margins identified to ensure that the entire lesion is removed) can result in prolonged and complete remission.

Surgery must take several criteria into account:

- Size and location of the sAVM
- Patient’s age
- Clinical classification (Schobinger): stage II is associated with a better prognosis and a less complicated/difficult surgery (Table 7).

**Table 7:** Study on surgery in AVMs

Reference	Type of study	Number of subjects	Age	Type of surgery	Efficacy	Tolerance
Goldenberg Dov et al. [34]	Prospective/ Cohort study	31	4–55	Surgical resection	Good	Good

Surgery of an AVM must involve a complete resection of the lesion, and requires:

- Complete nidus removal: emphasizing the importance of prior cross-sectional imaging and angiogram.
- Excision of the skin and often the periosteum adjacent to the AVM is usually necessary.
- A distinction must be made between the area of the nidus (requiring total excision) and peripheral areas with blood vessel recruitment, which may be spared and left in place.
- Unlike oncological surgery, histological examination does not provide insight into the quality of the excision.
- The surgeon's experience is crucial
  - Embolization 48–72 h before surgery if necessary, depending on the usual practices of the surgical teams.
  - Assessment prior to surgery of possible bleeding risks. Confirm that there are no coagulation disorders. Transfusions must be available for procedures with a bleeding risk. Certain new surgical techniques for hemostasis seem to be very interesting for the control of bleeding (e.g. harmonic scalpel).
  - Cervico-facial reconstruction using classic techniques ranging from the simplest to the most complicated: direct closure, local flaps, rarely skin grafting, microanastomosed flaps.

When total excision is impossible or would result in unacceptable sequelae, the following options are available:

- Repeated embolizations in the event of hemorrhage, which can sometimes lead to complications (tissue necrosis, superinfection, recurrence of hemorrhage).
- Rescue And/ or salvage surgery in the event of local complications (extensive necrosis) or even life-threatening hemorrhagic risks, despite or exacerbated by embolizations.
- In selected cases, after multidisciplinary discussion, planned partial excisions are sometimes proposed, for example in the case of cutaneous trophic disorders with a major risk of necrosis or hemorrhage, or for certain less active sAVMs whose localization would lead to unacceptable sequelae in the case of complete excision surgery.

In cases of non-surgical sAVMs due to their extensive nature or associated trophic disorders, amputation may be a reasonable option, especially at the extremities, after other alternatives have failed.

In summary, the main surgical scenarios are:

- Complete resection and straightforward reconstruction with few or no adverse effects: surgery is performed before puberty or in adulthood, depending on the expert teams, without prior embolization required, except in certain localizations.
- Complete resection is possible with a recurrence/progression risk, but there is a risk of reconstruction difficulties in the event of disease progression: surgery will be performed after informed consent and decision with the patient.
- Emergency surgery may be required for necrosis, infections (e.g. post-embolization), sAVMs during inflammatory flare-ups.
- sAVMs that cannot be completely removed, but are at stage III (Schobinger) or represent a major trophic risk: partial excision surgery of the most active, at-risk areas, often with flap reconstruction.

#### Surgical contraindications

Several situations may lead to the temporary or permanent contraindication of sAVM excision surgery:

- During episodes of exacerbation of a sAVM, surgery is often contraindicated or delayed due to surgical excision challenges or the risk of early recurrence.
- Some superficial sAVMs are too large and/or located in areas with a high risk of threat to the patient’s life, leading to a recommendation of simple monitoring in the absence of trophic disorders or complications.
- General condition: the patient's overall health may contraindicate a surgical procedure, particularly if there is a high risk of intraoperative bleeding.
- Classically, surgery is contraindicated when it can be predicted in advance that total excision is impossible.

Surgery is usually not proposed when excision will result in esthetic and/or functional sequelae more severe than the patient’s clinical preoperative status. The benefit/risk ratio must be assessed by a specialized multidisciplinary team and discussed with the patient.

This approach, however, is open for discussion: in some cases, excisions surgeries are suggested due to the risk of the sAVM progressing, which, due to its location and extension, can make excision even more complicated or lead to even greater sequelae. For instance, a superficial AVM affecting half of the lip but not reaching the labial commissure of the mouth may have a risk of causing disfigurement and more pronounced sequelae if it progresses to the labial commissure.

In the event of delayed healing, patients should be referred back to their care team to set up a protocol for wound dressings and skin care.

### Interventional radiology

The decision to perform endovascular treatment approach in an interventional radiology department is a multidisciplinary one, and must be taken on a case-by-case basis for each individual patient.

Embolization (endovascular navigation or direct puncture of the nidus) can be necessary in the following situations:

- Preoperatively (in rare cases) to reduce intraoperative bleeding
- Combined or in conjunction with surgical resection: the aim in this case is to treat part of the sAVM by embolization in order to avoid having to remove that part, thereby making the surgical unit to be removed smaller, leading to a more "harmonious" result,
- When it is the only therapeutic option,
- In case of emergency hemorrhagic complications.

Once a clear treatment plan and a specific goal of the interventional session have been defined, there are two types of approaches:

- Endovascular approach by means of microcatheter navigation: either transarterial or transvenous in selected cases with dominant venous outflow.
- Pecutaneous approach: always under diagnostic angiographic control, with direct puncture of the nidus and possibly a retrograde approach to the lesion via the venous route.
  o Thanks to the improved navigability of microcatheters, direct punctures are less common today. However, they may be a solution in AVMs depending on their angioarchitecture type and AVM location. The goal may then be solely to access the nidus and occlude specific locations. In emergency, they offer the advantage of targeting the lesions directly.

The choice is based on:

- The anatomical location of the AVM: cervicofacial, limb, thoracic or visceral location;
- Tissue invasion by AVMs: cutaneous, subcutaneous, muscular, osseous or visceral involvement;
- Angioarchitecture and flow velocity visualized on arteriogram
- The second step is the choice of embolization material, based on the above-mentioned criteria.
- Ideally, in a cervicofacial location, a resorbable, liquid embolizing agent that does not stain the skin (e.g. 98% ethanol) should be used. Care should be taken to monitor the skin's appearance after embolization, given the risk of skin necrosis or post-operative paresis due to alcoholization of a nerve branch.
- In a deeper location, other embolization materials that are less fluid, partially resorbable, and have a more controlled injection can be considered, for example: Onyx®, PHIL®, Histoacryl®, Glubran®2, Bleomycin, etc.

Liquid embolic agents such as glue (n-butyl cyanoacrylate), ethylene vinyl alcohol (EVOH) or 2-poly-hydroxyethyl methacrylate (pHEMA) are preferred. Each material has its own advantages and disadvantages. Glue and PHEMA have the advantage of not leaving a tattoo effect on the skin, as can happen with the tantalum used to make EVOH radiopaque [15].

The choice of embolization material must take into account the additional examinations required for monitoring, and must not complicate their interpretation (artifact) or make 2nd-line surgery difficult (Onyx® and coils).

In some cases, venous access routes have been described with occlusion of the drainage pathway and the use of coils and plugs, in addition to retrograde embolization of the nidus.

All these treatments are usually performed under general anesthetic and under fluoroscopic control by a trained operator.

**Fig. 4:**
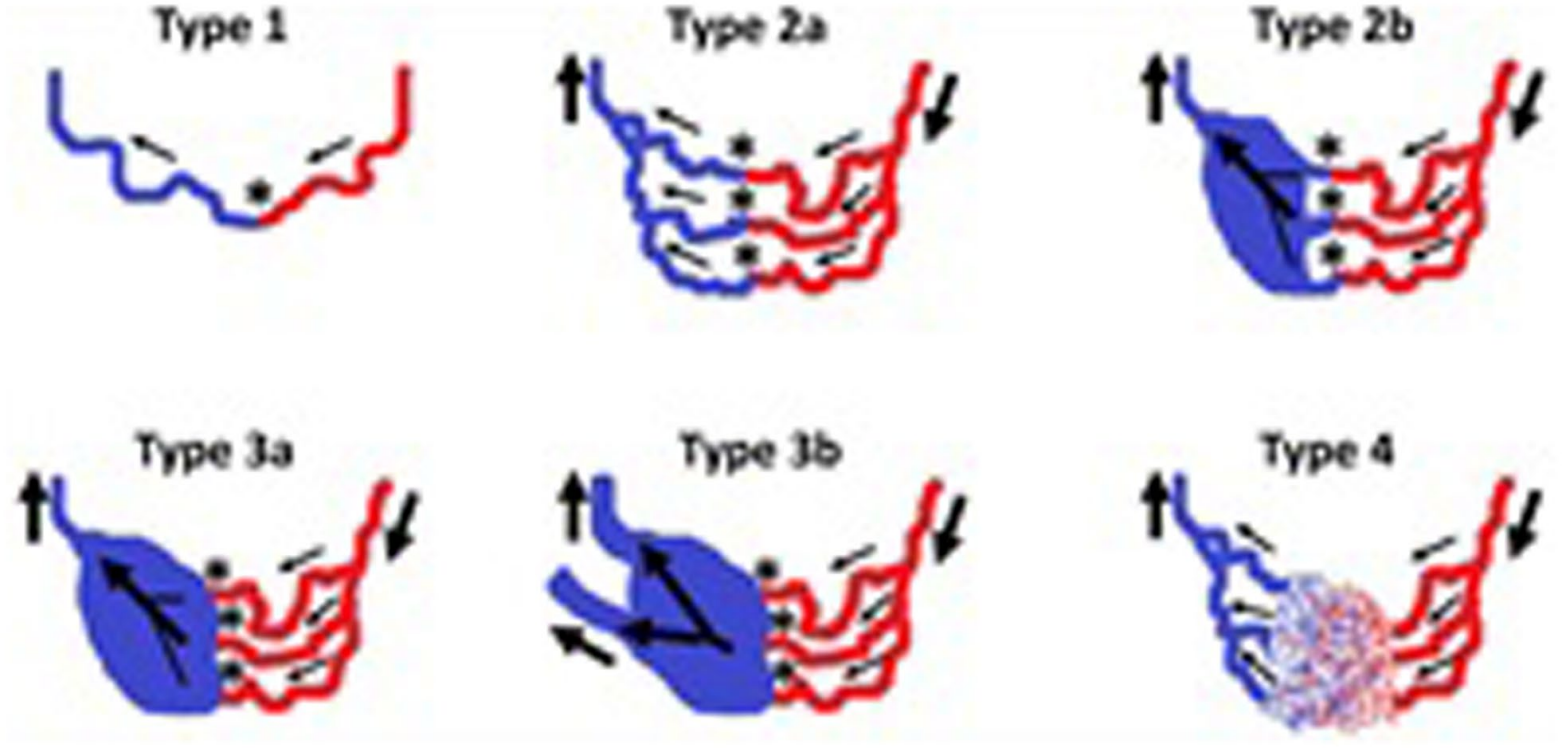
Yakes classification of AVM angioarchitecture. Figure is extracted from Massoni L et al. 2021 (Fig. 5) [36]. Type 1: direct single arteriovenous shunt called arteriovenous fistulae (AVF), Type 2a:  Multiple arteries/arterioles directly connected to outflow drainage veins into a typical “nidus”, Type 2b: Same as Type 2a except that the "nidus" drains into l a single aneurysmal drainage vein, Type 3a: Multiple inflow arteries/arterioles shunting into a single aneurysmal drainage vein. AV shunts are in a vein-wall aneurysm**,**Type 3b: Multiple inflow arterioles shunting into a single aneurysmal drainage vein, that connect to multiple outflow veins. AV shunts are in a vein-wall aneurysm, Type 4: Multiple inflow transversing arteries and arterioles and microfistulas shunting with  multiple outflow veins

The Yakes classification is currently often cited when deciding on the approach to take for endovascular treatment [35] (Fig. 4, Table 8).

**Table 8:** Studies on imaging and endovascular treatment in AVMs

References	Type of study	Number of subjects	Age	Device used	Efficacy	Tolerance
Dobson et al. [37]	Prospective / cohort study	14	8–51	Magnetic resonance angiography and MRI	13/14	Good
Kazutomo Nakazawa et al. [38]	Prospective	1	50	Transarterial embolization	Good	Good
Su et al. [39]	Prospective / cohort study	168	16–46	Ethanol embolotherapy	56/66	Good
Saeed Kilani et al. [40]	Prospective / cohort study	19	2–72	Onyx embolization	12/19	Good
Park et al. [41]	Prospective / cohort study	306	1–74	Endovascular treatment	54.2% and 64.3%	Good
Jin et al. [42]	Prospective / cohort study	32	2–60	Intralesional interstitial injection of bleomycin	27/32	Good
Kitagawa et al. [43]	Prospective cohort study	23	4–74	Polidocanol sclerotherapy combined with transarterial embolization using a liquid adhesive agent (n-butyl cyanoacrylate)	20/23	Good

### Clinical follow-up

Monitoring is essentially clinical and adapted to the severity of symptoms. The frequency varies from every 3 months to once a year, or even every 2 years. It is more frequent during periods of hormonal change (puberty, pregnancy). Given the difficulties involved in treating AVMs and, above all, the absence of a simple, effective, codified treatment, management is almost always the result of multidisciplinary, personalized follow-up and consideration for each patient.

It requires a therapeutic strategy, frequently involving the successive or parallel combination of several therapeutic measures.

- Skin: As a natural protective barrier, care must be taken to ensure that it is not altered by dilatation of subcutaneous veins due to AV shunting and increased flow or hemodiversion/vascular steal syndrome. Compression is essential, and must be well adapted to avoid skin erosion.
- Functionality of the limb: ensure that there is no muscular or skeletal impact. Flexion contractures or limb impairment may occur due to musculoskeletal or joint involvement. Lower limb length discrepancy may develop as a result of excessive growth, leading to effects on the spine in the form of scoliosis. A specialized orthopedic consultation may be recommended.
- Vessels: monitoring of subcutaneous venous or arterial aneurysms at risk, both clinically and by Doppler ultrasound. Combined with clinical monitoring, repeated measurement of comparative flow rates enables monitoring of the evolution of sAVMs. WSS (Wall Shear Stress) may be a new marker for assessing the prognosis of sAVMs.
- General impact: signs of heart output failure (fatigue, dyspnea, lower limb edema).
- Psychological impact: this needs to be assessed for the patient and their caregivers at each consultation.

### Lifestyle advice and therapeutic patient education

#### Physical activities

Lifestyle must be adapted to the pathology and its location. Any local trauma should be avoided, as can occur in contact sports or certain high-risk occupations. There are no absolute prohibitions, but preferences should be discussed in a multidisciplinary consultation to assist the patient in making life choices. In the case of children, they should be guided as early as possible towards a low-impact sport (e.g. swimming).

Therapeutic patient education (TPE) also consists of warning patients of high-risk situations. There is currently no specific TPE program dedicated to people with AVMs. TPE projects dedicated to AVMs are planned in France for 2024.

#### Contraception

Estrogen-based contraception is contraindicated, as it may induce a progressive exacerbation of AVMs. There are no particular restrictions on other types of contraception.

#### Pregnancy

Pregnancy is not contraindicated, but needs to be anticipated and carefully monitored by the specialized multidisciplinary team. Progressive aggravation of sAVMs has been observed during/after pregnancy in some patients.

Studies on the impact of hormonal changes on sAVMs: no publications to date.

#### Lifestyle

A healthy diet is essential to avoid excess weight, particularly in the case of limb sAVMs.

### Psychological management

sAVMs can cause considerable upheaval in the lives of patients and their families, particularly at the time of diagnosis and when the disease progresses. They are also faced with lifestyle restrictions.

Patients and/or their caregivers may need occasional psychological support; this varies from one person to another, depending on the events they have experienced, their age and the progression of their disease.

Patients and caregivers should be offered regular psychological support (e.g. psychologist from the multidisciplinary medical team). Follow-up is carried out in collaboration with the treating physician.

Studies on the psychological impact of AVMs: No publications to date.

### Social care

Superficial AVMs can lead to various kinds of social difficulties. Patients/parents can turn to their treating physician, to a specialist on the hospital's multidisciplinary team, to the hospital's social services department or to patient associations for guidance in accessing the appropriate social services and advice on administrative procedures and patient rights.

#### Support from patient organizations

In France: the patient organization “Super MAVs” is currently being created.

In Belgium: Vascapa www.vascapa.org

In Netherland:

- Hevas—Patiëntenvereniging voor hemangiomen en vasculaire malformaties https://www.hevas.eu/
- CMTC-OVN: https://www.cmtc.nl/en/

Lists of patient organizations by disease and country can be found on Orphanet.

## Conclusions

Care for Superficial Arteriovenous Malformations (sAVM) requires caution and expertise. Their management is always complex and requires multidisciplinary consultation. The natural history is characterized by either a gradual increase in size or intermittent exacerbations triggered by hormonal changes or trauma. Therapeutic strategies may include clinical follow-up, compression garments, embolization and/or excision surgery and drug therapies. Psychosocial issues also need to be considered.

## Data Availability

Not applicable.

## Abbreviations

AVF: Arteriovenous fistula
AVM: Arteriovenous malformation
CM-AVM: Capillary malformation-arteriovenous malformation
CMPP: *Centre Médico Psychologique Pédiatrique* (Pediatric Medical and Psychological Center)
CT scan: Computed tomography scan
ENT: Ear, nose and throat (ENT) surgery (Otorhinolaryngology)
FGF: Fibrobalst growth factor
HAS: *Haute Autorité de Santé* (French Health Authority)
ISSVA: International Society for the Study of Vascular Anomalies
MA: Marketing authorization
MAPK: Mitogen activated protein kinase
MRI: Magnetic resonance imaging
ORL: Otorhinolaryngology
PDGF: Platelet derived growth factor
PNDS: *Protocole National de Diagnostic et de Soins* (National Diagnostic and Care Protocol)
sAVM: Superficial arteriovenous malformation
TPE: Therapeutic patient education
TNF: Tumor necrosis factor
WSS: Wall shear stress
